# Supplementary material for: Mutations and Copy Number Alterations in IDH Wild-Type Glioblastomas Are Shaped by Different Oncogenic Mechanisms
Source: Biomedicines. 2020 Dec 7;8(12):574. doi: 10.3390/biomedicines8120574 (PMC7762325; doi:10.3390/biomedicines8120574)
Supplement: Supplementary file 1 [file biomedicines-08-00574-s001.zip › biomedicines-966335 supplementary/Table_S1.docx]

**Supplementary Table 1. Coverage statistics for all samples analyzed in the study.** “Sample ID” indicates the “blood” or “tumor” sample. “mean” indicates the mean coverage. “>1X”, “>5X”, “>10X”, “>25X”, “>50X” and “>100X” indicate percentages of reads with at least 1, 5, 10, 25, 50 and 100-fold coverage, respectively.

| **Sample ID** | **Exome capture kit** | **mean** | **>1X** | **>5X** | **>10X** | **>25X** | **>50X** | **>100X** |
| --- | --- | --- | --- | --- | --- | --- | --- | --- |
| NOT-0046-blood | SeqCap EZ MedExome | 92.7 | 100 | 99.9 | 99.6 | 98 | 75.8 | 29.4 |
| NOT-0046-tumorA | SeqCap EZ MedExome | 66.01 | 100 | 99.9 | 99.5 | 91.7 | 59.7 | 13.2 |
| NOT-0046-tumorB | SeqCap EZ MedExome | 203.88 | 100 | 99.9 | 99.9 | 99.6 | 97.7 | 78.9 |
| NOT-0047-blood | SeqCap EZ Exome v2 | 108.24 | 99.9 | 99.3 | 98.4 | 93.3 | 78.1 | 44.8 |
| NOT-0047-tumor | SeqCap EZ Exome v2 | 197.19 | 99.9 | 99.6 | 99.1 | 97.1 | 90.9 | 72.1 |
| NOT-0048-blood | SeqCap EZ Exome v2 | 97.4 | 99.7 | 99 | 97.9 | 91.6 | 73.8 | 39.1 |
| NOT-0048-tumor | SeqCap EZ Exome v2 | 204.16 | 99.8 | 99.5 | 99 | 97.2 | 91.8 | 74.1 |
| NOT-0051-blood | SeqCap EZ MedExome | 79.06 | 100 | 99.8 | 99.5 | 95.6 | 67.2 | 19.8 |
| NOT-0051-tumor | SeqCap EZ MedExome | 140.56 | 100 | 99.9 | 99.8 | 99.3 | 95 | 59 |
| NOT-0052-blood | SeqCap EZ MedExome | 52.69 | 99.6 | 98.9 | 96.4 | 77.5 | 38.2 | 7.6 |
| NOT-0052-tumor | SeqCap EZ MedExome | 109.19 | 99.7 | 99.6 | 99.4 | 98.1 | 83.4 | 38 |
| NOT-0054-blood | SeqCap EZ MedExome | 77.55 | 99.7 | 99.5 | 99.2 | 94.9 | 64 | 19.4 |
| NOT-0054-tumor | SeqCap EZ MedExome | 171.93 | 99.7 | 99.7 | 99.6 | 99.1 | 96.2 | 70.4 |
| NOT-0056-blood | SeqCap EZ MedExome | 111.13 | 99.7 | 99.6 | 99.5 | 98.7 | 88.3 | 42.1 |
| NOT-0056-tumor | SeqCap EZ MedExome | 116.53 | 99.7 | 99.6 | 99.5 | 98.4 | 87.5 | 45 |
| NOT-0057-blood | xGen Exome Research Panel | 181.86 | 99.8 | 99.8 | 99.7 | 99.5 | 98.5 | 86.4 |
| NOT-0057-tumor | xGen Exome Research Panel | 154.71 | 99.9 | 99.8 | 99.7 | 99.4 | 96.9 | 74.5 |
| NOT-0058-blood | xGen Exome Research Panel | 43.89 | 99.7 | 99.4 | 98.3 | 81.8 | 30.9 | 2.3 |
| NOT-0058-tumor | xGen Exome Research Panel | 78.19 | 99.7 | 99.7 | 99.4 | 95.9 | 74.2 | 22.2 |
| NOT-0059-blood | xGen Exome Research Panel | 108.54 | 99.8 | 99.7 | 99.6 | 98.7 | 91.4 | 49 |
| NOT-0059-tumor | xGen Exome Research Panel | 218.44 | 99.8 | 99.8 | 99.7 | 99.6 | 98.8 | 91 |
| NOT-0060-blood | xGen Exome Research Panel | 288.45 | 99.9 | 99.9 | 99.9 | 99.8 | 99.4 | 96.3 |
| NOT-0060-tumor | xGen Exome Research Panel | 167.87 | 99.9 | 99.9 | 99.9 | 99.6 | 97.8 | 82.3 |
| NOT-0061-blood | xGen Exome Research Panel | 77.64 | 99.7 | 99.3 | 98.8 | 95.1 | 75.5 | 22 |
| NOT-0061-tumor | xGen Exome Research Panel | 138.2 | 99.9 | 99.7 | 99.5 | 98.7 | 95.3 | 71.5 |
| NOT-0062-blood | xGen Exome Research Panel | 85.33 | 99.7 | 99.3 | 98.7 | 96 | 81.3 | 29.9 |
| NOT-0062-tumor | xGen Exome Research Panel | 199.36 | 99.9 | 99.8 | 99.7 | 99.1 | 97.1 | 84.4 |
| NOT-0063-blood | xGen Exome Research Panel | 109.53 | 99.9 | 99.8 | 99.7 | 98.4 | 90.5 | 51.4 |
| NOT-0063-tumor | xGen Exome Research Panel | 184.08 | 99.9 | 99.9 | 99.8 | 99.4 | 97.5 | 84.5 |
| NOT-0064-blood | xGen Exome Research Panel | 118.94 | 99.7 | 99.6 | 99.4 | 98 | 89.8 | 55.8 |
| NOT-0064-tumor | xGen Exome Research Panel | 246.45 | 99.7 | 99.7 | 99.6 | 99.4 | 98.2 | 91 |
| NOT-0065-blood | xGen Exome Research Panel | 218.79 | 99.9 | 99.8 | 99.7 | 99.3 | 97.7 | 88.9 |
| NOT-0065-tumor | xGen Exome Research Panel | 156.89 | 99.8 | 99.6 | 99.4 | 98.4 | 94.6 | 75.4 |
| NOT-0066-blood | xGen Exome Research Panel | 188.28 | 99.6 | 99.3 | 99 | 98.2 | 95.9 | 84.6 |
| NOT-0066-tumor | xGen Exome Research Panel | 263.48 | 99.7 | 99.6 | 99.5 | 99.1 | 97.7 | 91.1 |
| NOT-0067-blood | xGen Exome Research Panel | 147.78 | 99.7 | 99.7 | 99.6 | 99.1 | 97 | 78.1 |
| NOT-0067-tumor | xGen Exome Research Panel | 220.14 | 99.8 | 99.7 | 99.7 | 99.4 | 98.2 | 89.7 |
| NOT-0068-blood | xGen Exome Research Panel | 114.33 | 99.9 | 99.8 | 99.7 | 98.5 | 91 | 53.2 |
| NOT-0068-tumor | xGen Exome Research Panel | 246.44 | 99.9 | 99.9 | 99.8 | 99.6 | 98.6 | 91.8 |
| NOT-0069-blood | xGen Exome Research Panel | 128.98 | 99.9 | 99.9 | 99.8 | 99 | 94.8 | 65.5 |
| NOT-0069-tumorA | xGen Exome Research Panel | 230.28 | 99.9 | 99.9 | 99.8 | 99.5 | 98.1 | 89.1 |
| NOT-0069-tumorB | xGen Exome Research Panel | 240.51 | 99.9 | 99.9 | 99.9 | 99.7 | 98.8 | 93.3 |
| NOT-0070-blood | xGen Exome Research Panel | 140.56 | 99.9 | 99.8 | 99.7 | 98.9 | 95.2 | 71 |
| NOT-0070-tumor | xGen Exome Research Panel | 130.41 | 99.9 | 99.9 | 99.7 | 98.6 | 88.8 | 55.6 |
| NOT-0071-blood | xGen Exome Research Panel | 103.57 | 99.9 | 99.9 | 99.8 | 98.7 | 90.1 | 45.4 |
| NOT-0071-tumor | xGen Exome Research Panel | 174.57 | 99.9 | 99.9 | 99.9 | 99.6 | 97.7 | 80.8 |
| NOT-0073-blood | xGen Exome Research Panel | 114.48 | 99.9 | 99.8 | 99.7 | 98.8 | 94 | 58.8 |
| NOT-0073-tumor | xGen Exome Research Panel | 194.98 | 99.9 | 99.9 | 99.8 | 99.5 | 98.2 | 88.7 |
| NOT-0075-blood | xGen Exome Research Panel | 128.54 | 99.7 | 99.7 | 99.6 | 99.2 | 96.6 | 68.9 |
| NOT-0075-tumorA | xGen Exome Research Panel | 180.67 | 99.8 | 99.7 | 99.6 | 99.2 | 97.5 | 83.7 |
| NOT-0075-tumorB | xGen Exome Research Panel | 306.02 | 99.8 | 99.7 | 99.7 | 99.5 | 98.8 | 93.7 |
| NOT-0076-blood | xGen Exome Research Panel | 106.8 | 99.9 | 99.9 | 99.8 | 98.9 | 91.3 | 48.1 |
| NOT-0076-tumor | xGen Exome Research Panel | 186.04 | 99.9 | 99.9 | 99.9 | 99.6 | 97.8 | 83.9 |
| NOT-0078-blood | xGen Exome Research Panel | 92.92 | 99.7 | 99.7 | 99.6 | 98.5 | 87.6 | 34.2 |
| NOT-0078-tumor | xGen Exome Research Panel | 174.4 | 99.8 | 99.7 | 99.7 | 99.5 | 98.6 | 86.9 |
| NOT-0079-blood | xGen Exome Research Panel | 104.68 | 99.9 | 99.9 | 99.8 | 98.7 | 90.2 | 46.7 |
| NOT-0079-tumor | xGen Exome Research Panel | 253.28 | 99.9 | 99.9 | 99.9 | 99.7 | 98.8 | 92.2 |
| NOT-0082-blood | xGen Exome Research Panel | 118.24 | 99.9 | 99.9 | 99.8 | 99.1 | 93.1 | 57.1 |
| NOT-0082-tumor | xGen Exome Research Panel | 184.25 | 100 | 99.9 | 99.9 | 99.8 | 98.2 | 68.3 |
| NOT-0083-blood | xGen Exome Research Panel | 110.83 | 99.8 | 99.7 | 99.6 | 99 | 92.8 | 52.2 |
| NOT-0083-tumor | xGen Exome Research Panel | 225.79 | 99.8 | 99.7 | 99.7 | 99.6 | 98.5 | 88.3 |
| NOT-0084-blood | xGen Exome Research Panel | 144.66 | 99.9 | 99.9 | 99.8 | 99.5 | 96.9 | 74.8 |
| NOT-0084-tumor | xGen Exome Research Panel | 296.57 | 99.9 | 99.9 | 99.9 | 99.4 | 97.3 | 87.8 |
| NOT-0085-blood | xGen Exome Research Panel | 178.76 | 99.9 | 99.9 | 99.9 | 99.7 | 98.3 | 85.6 |
| NOT-0085-tumor | xGen Exome Research Panel | 330.89 | 99.9 | 99.9 | 99.9 | 99.9 | 99.6 | 97.7 |
| NOT-0086-blood | xGen Exome Research Panel | 187.39 | 99.9 | 99.9 | 99.9 | 99.7 | 98.6 | 88.1 |
| NOT-0086-tumor | xGen Exome Research Panel | 278.96 | 99.9 | 99.9 | 99.9 | 99.7 | 99.3 | 95.9 |
| NOT-0087-blood | xGen Exome Research Panel | 203.09 | 99.9 | 99.9 | 99.9 | 99.7 | 98.6 | 89 |
| NOT-0087-tumor | xGen Exome Research Panel | 248.64 | 99.9 | 99.9 | 99.9 | 99.7 | 99 | 93.4 |
| NOT-0088-blood | xGen Exome Research Panel | 111.13 | 99.7 | 99.7 | 99.6 | 99 | 93.3 | 52.3 |
| NOT-0088-tumorA | xGen Exome Research Panel | 234.93 | 99.8 | 99.7 | 99.7 | 99.5 | 98.8 | 93.9 |
| NOT-0088-tumorB | xGen Exome Research Panel | 210.59 | 99.8 | 99.7 | 99.7 | 99.5 | 98.6 | 90.7 |
| NOT-0089-blood | xGen Exome Research Panel | 116.03 | 99.9 | 99.8 | 99.7 | 98.9 | 92.8 | 54.7 |
| NOT-0089-tumor | xGen Exome Research Panel | 233.34 | 99.9 | 99.9 | 99.8 | 99.6 | 98.9 | 92.3 |
| NOT-0090-blood | xGen Exome Research Panel | 161.03 | 99.9 | 99.9 | 99.9 | 99.4 | 96.3 | 75.1 |
| NOT-0090-tumorA | xGen Exome Research Panel | 228.47 | 99.9 | 99.9 | 99.9 | 99.7 | 99 | 92.6 |
| NOT-0090-tumorB | xGen Exome Research Panel | 302.96 | 99.9 | 99.9 | 99.9 | 99.8 | 99.4 | 96.1 |
| NOT-0091-blood | xGen Exome Research Panel | 131.7 | 99.8 | 99.7 | 99.7 | 99.3 | 96.2 | 66.8 |
| NOT-0091-tumor | xGen Exome Research Panel | 291.18 | 99.8 | 99.7 | 99.7 | 99.6 | 99.3 | 97.2 |
| NOT-0092-blood | xGen Exome Research Panel | 127.53 | 99.9 | 99.9 | 99.9 | 99.5 | 95.9 | 66.2 |
| NOT-0092-tumorA | xGen Exome Research Panel | 284.66 | 99.9 | 99.9 | 99.9 | 99.7 | 99.1 | 94.5 |
| NOT-0092-tumorB | xGen Exome Research Panel | 267.96 | 99.9 | 99.9 | 99.9 | 99.7 | 99.1 | 95.2 |
| NOT-0094-blood | xGen Exome Research Panel | 110.61 | 99.9 | 99.9 | 99.8 | 99.1 | 93.8 | 52.9 |
| NOT-0094-tumor | xGen Exome Research Panel | 153.22 | 99.9 | 99.9 | 99.8 | 99.3 | 96.1 | 74.3 |
