# Supplementary material for: Mutations and Copy Number Alterations in IDH Wild-Type Glioblastomas Are Shaped by Different Oncogenic Mechanisms
Source: Biomedicines. 2020 Dec 7;8(12):574. doi: 10.3390/biomedicines8120574 (PMC7762325; doi:10.3390/biomedicines8120574)
Supplement: Supplementary file 1 [file biomedicines-08-00574-s001.zip › biomedicines-966335 supplementary/Table_S2.docx]

**Supplementary Table 2. Putative double minute chromosomes detected in the study.** “ID” indicates the tumor in which the double minute chromosome was observed. “Chromosome” indicates the chromosome in which the double minute chromosome was observed. “Oncogenes identified within the double minute” lists the oncogenes identified within the double minute chromosome.

| ID | Chromosome | Oncogenes identified within the double minute |
| --- | --- | --- |
| NOT-0048 | chr21 | APP |
| NOT-0052 | chr1 | LRRN2, ELK4, MDM4, RBBP5, REN, NUCKS1, SLC45A3 |
| NOT-0060 | chr7 | EGFR, SEC61G, VOPP1 |
| NOT-0062 | chr12 | CDK4, AGAP2, MDM2 |
| NOT-0064 | chr4 | FGFR3, SLIT2 |
| NOT-0065 | chr7 | EGFR, SEC61G, PSPH, VOPP1, CDK6 |
| NOT-0066 | chr12 | DDIT3, MARS1, CDK4, AGAP2, HMGA2, MDM2 |
| NOT-0067 | chr1 | LRRN2, MDM4, ATP2B4, RBBP5, REN, SNRPE, SOX13 |
| NOT-0067 | chr7 | EGFR, SEC61G |
| NOT-0070 | chr3 | PIK3CA, ACTL6A, DCUN1D1, SOX2, FXR1 |
| NOT-0070 | chr12 | LRP1, SHMT2, AGAP2, DDIT3, DTX3, GLI1, KIF5A, MARS1, CDK4, MDM2 |
| NOT-0073 | chr7 | EGFR, SEC61G |
| NOT-0084 | chr7 | TES, MET, CAV1, CAV2, CFTR, WNT2 |
| NOT-0085 | chr7 | EGFR, SEC61G, VOPP1, PSPH |
| NOT-0087 | chr7 | EGFR, SEC61G |
| NOT-0091 | chr7 | MACC1, IL6, EGFR, SEC61G |
| NOT-0092_TA | chr19 | BRD4, CCNE1 |
| NOT-0094 | chr7 | EGFR, SEC61G, VOPP1 |
