# Supplementary figures and images for: Mutations and Copy Number Alterations in IDH Wild-Type Glioblastomas Are Shaped by Different Oncogenic Mechanisms
Source: Biomedicines. 2020 Dec 7;8(12):574. doi: 10.3390/biomedicines8120574 (PMC7762325; doi:10.3390/biomedicines8120574)

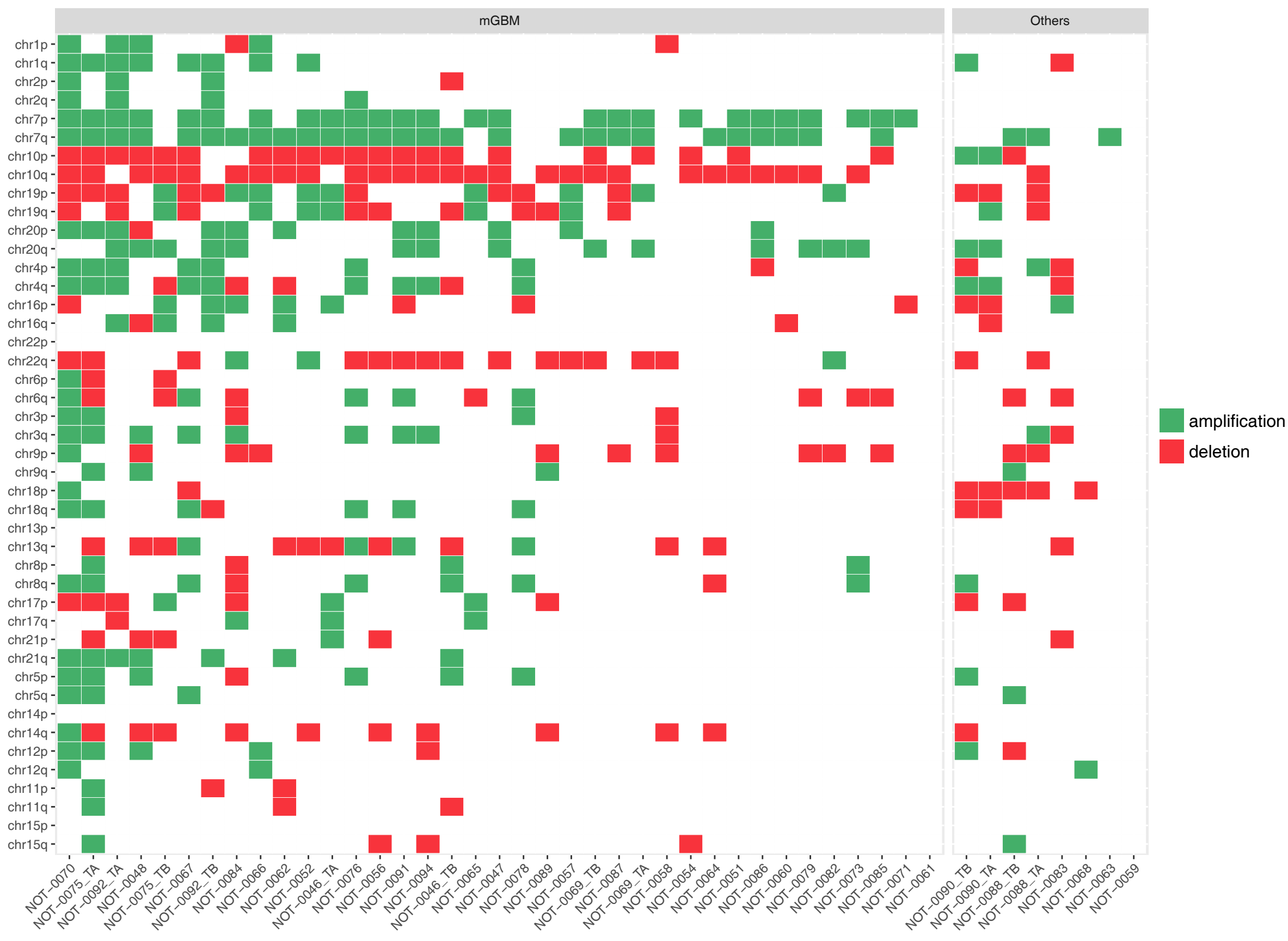

Supplement: Supplementary file 1 [file biomedicines-08-00574-s001.zip › biomedicines-966335 supplementary/Figure_S1.pdf]

mGBM (n = 31)

A

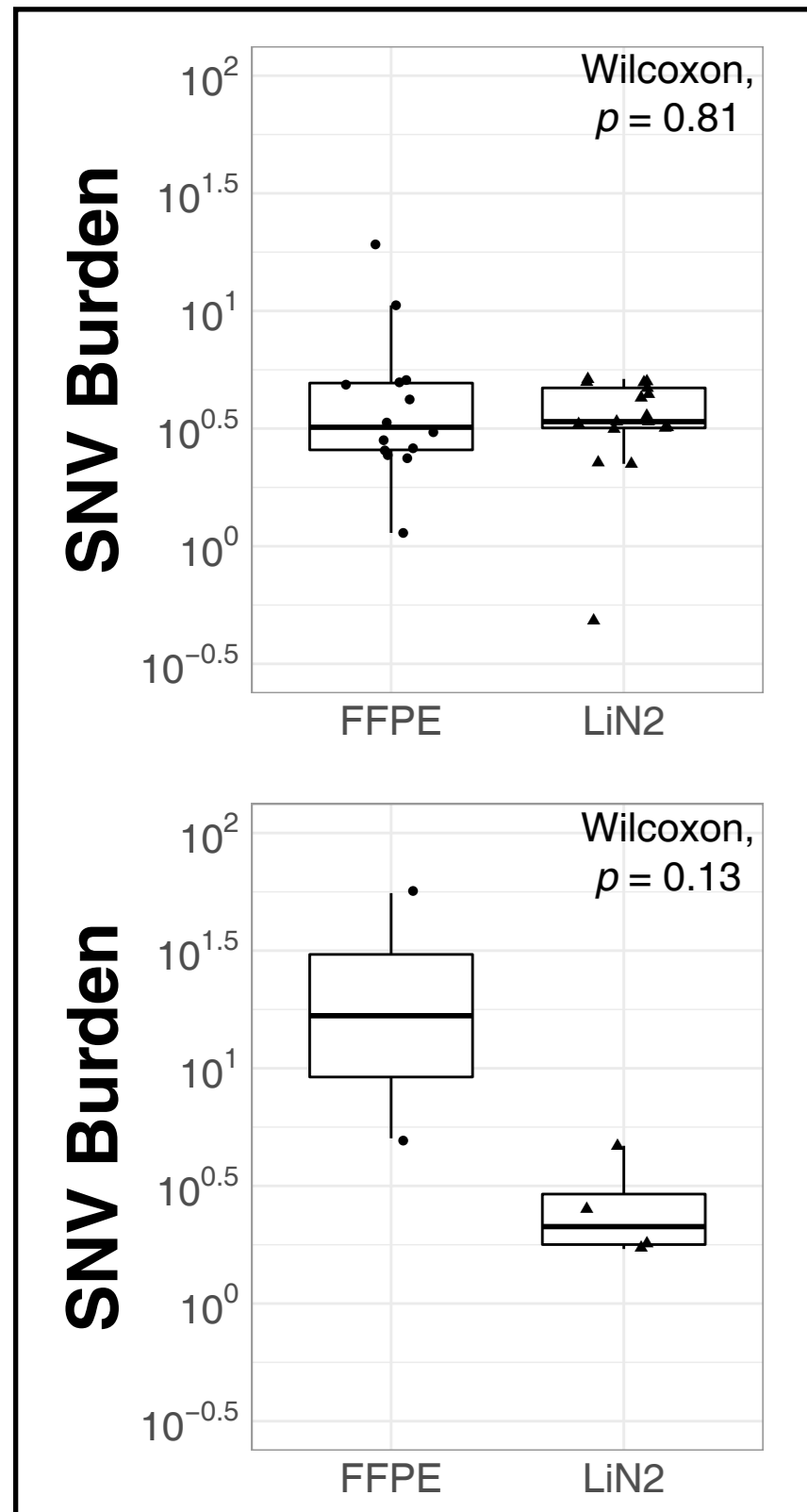

B

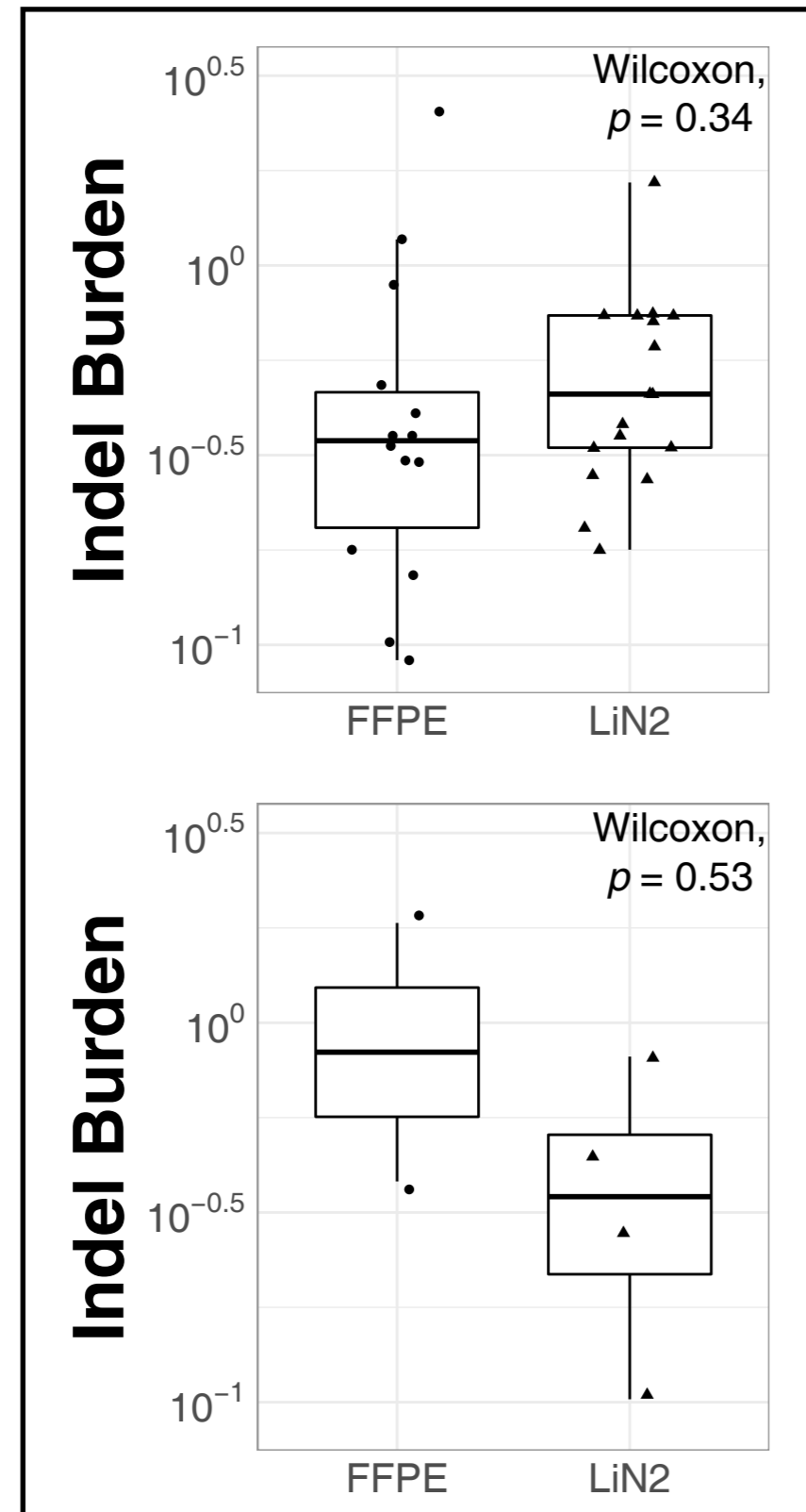

C

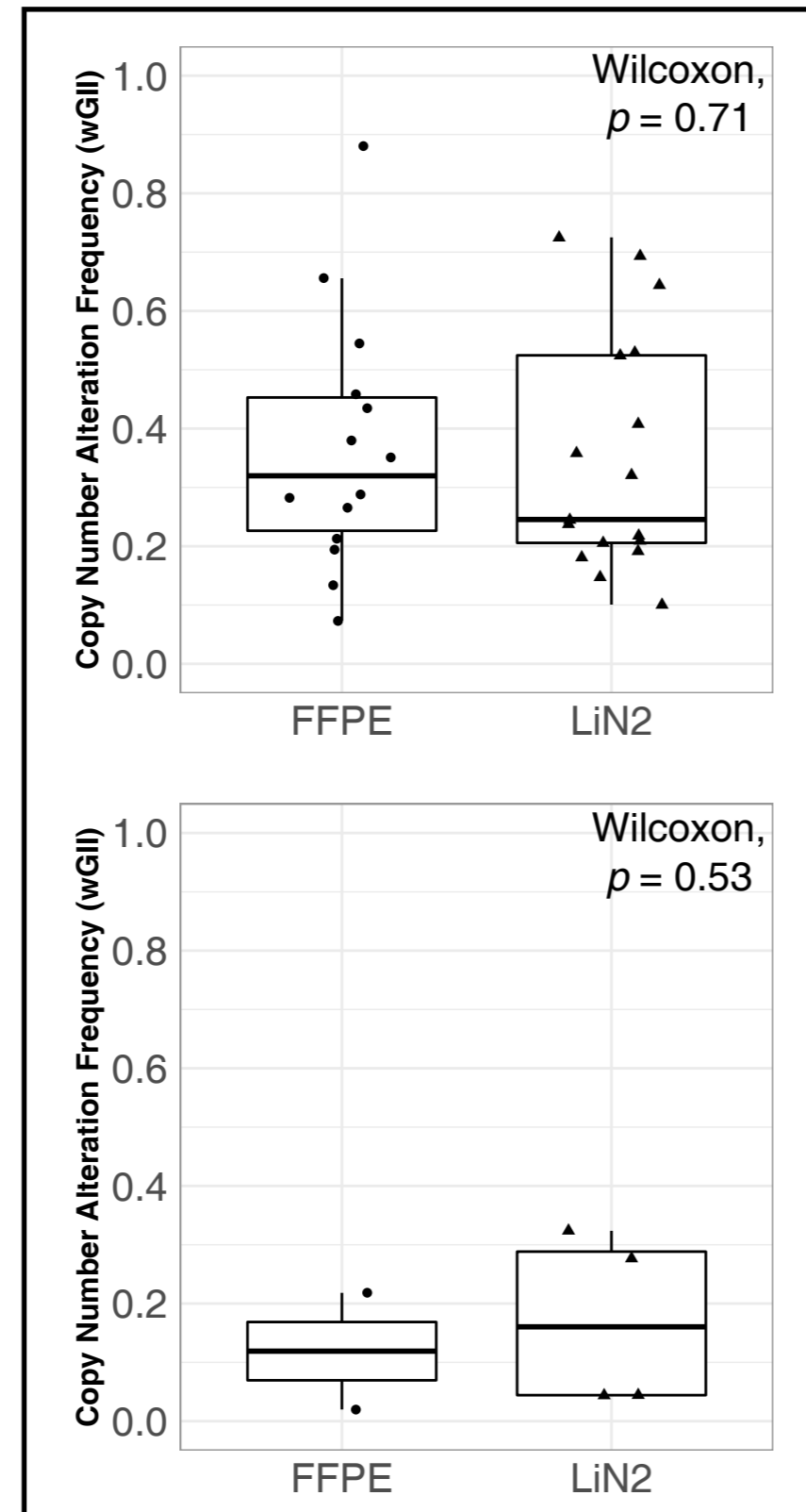

D

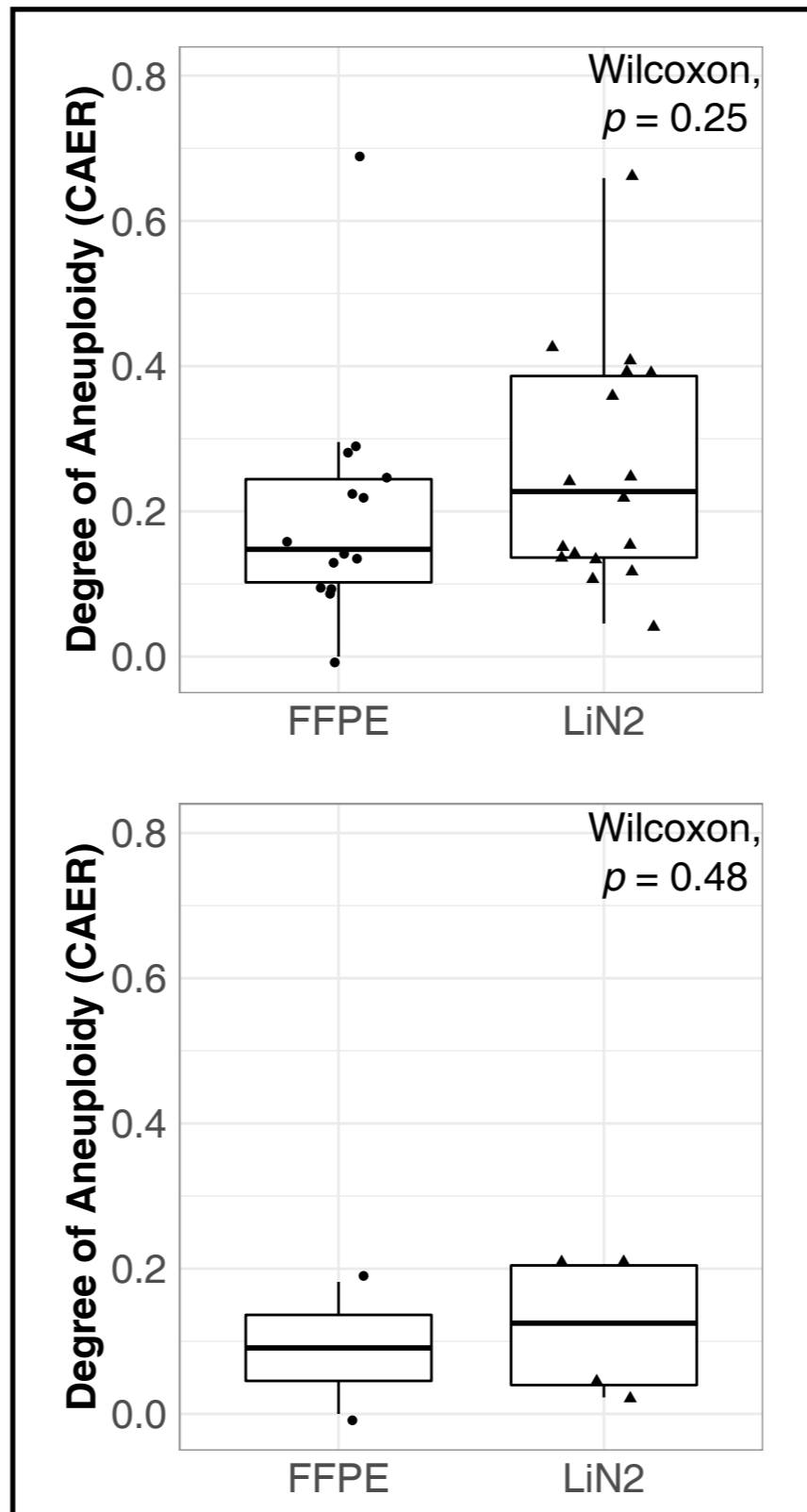

E

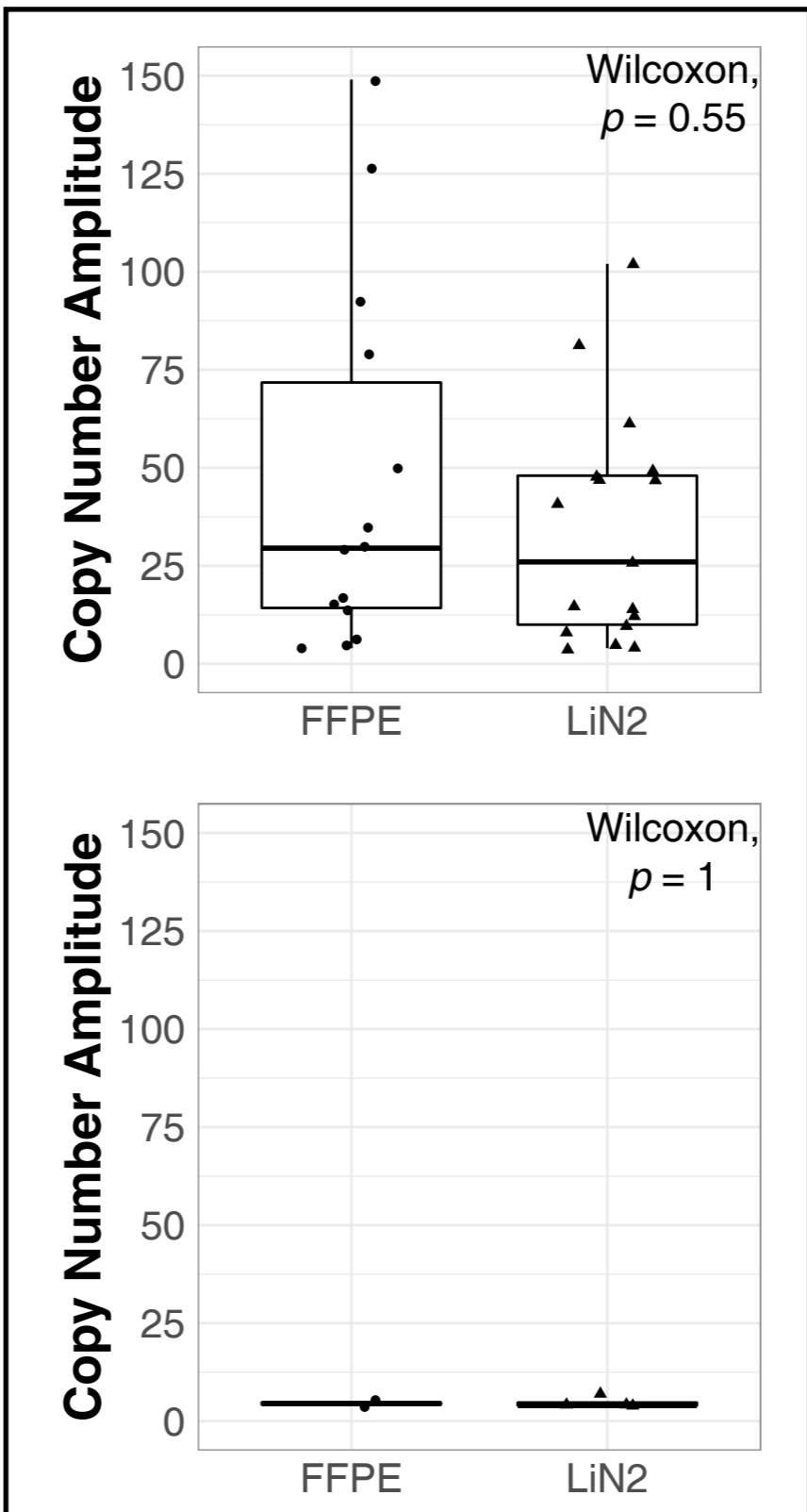

F

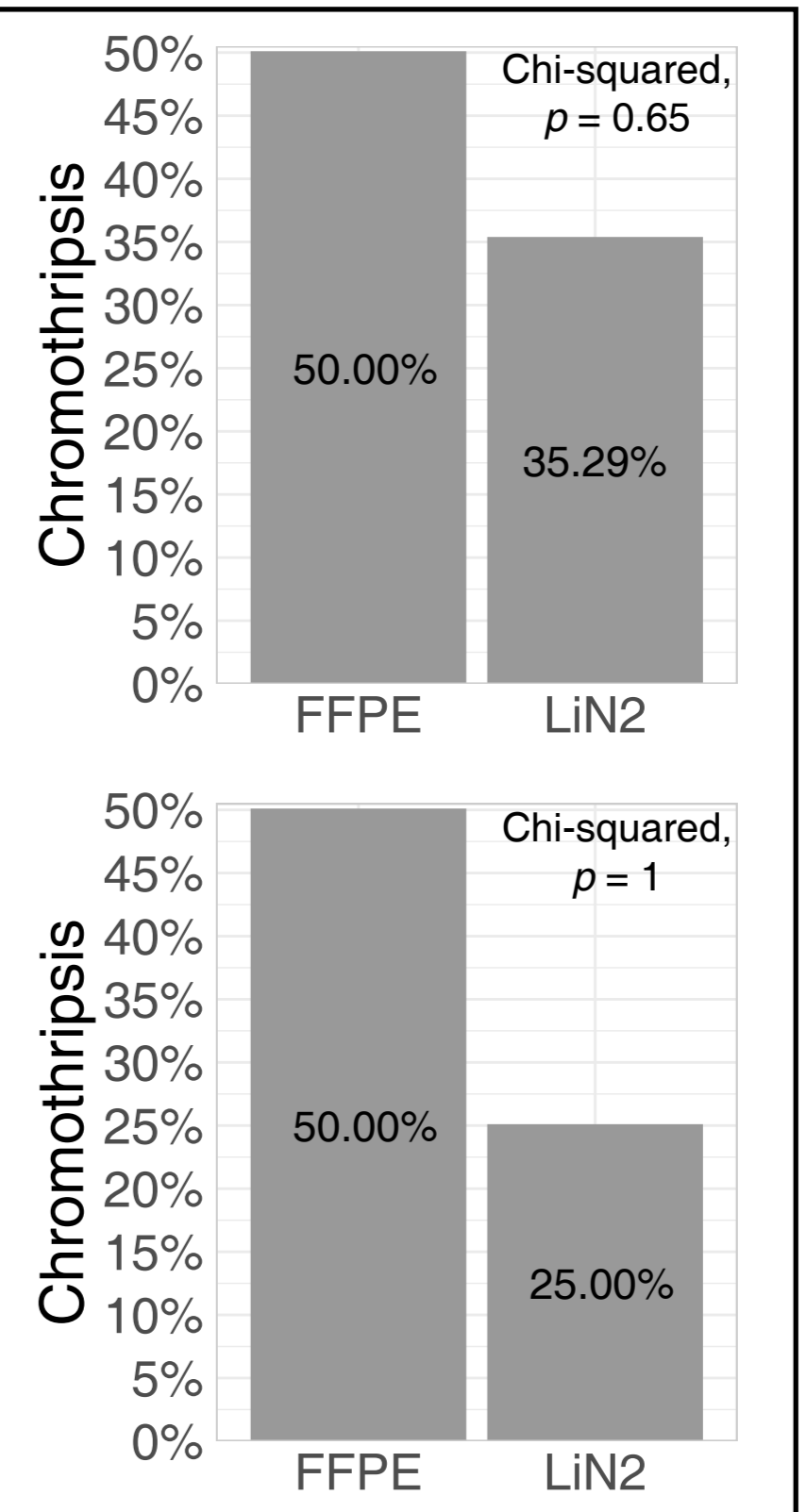

G

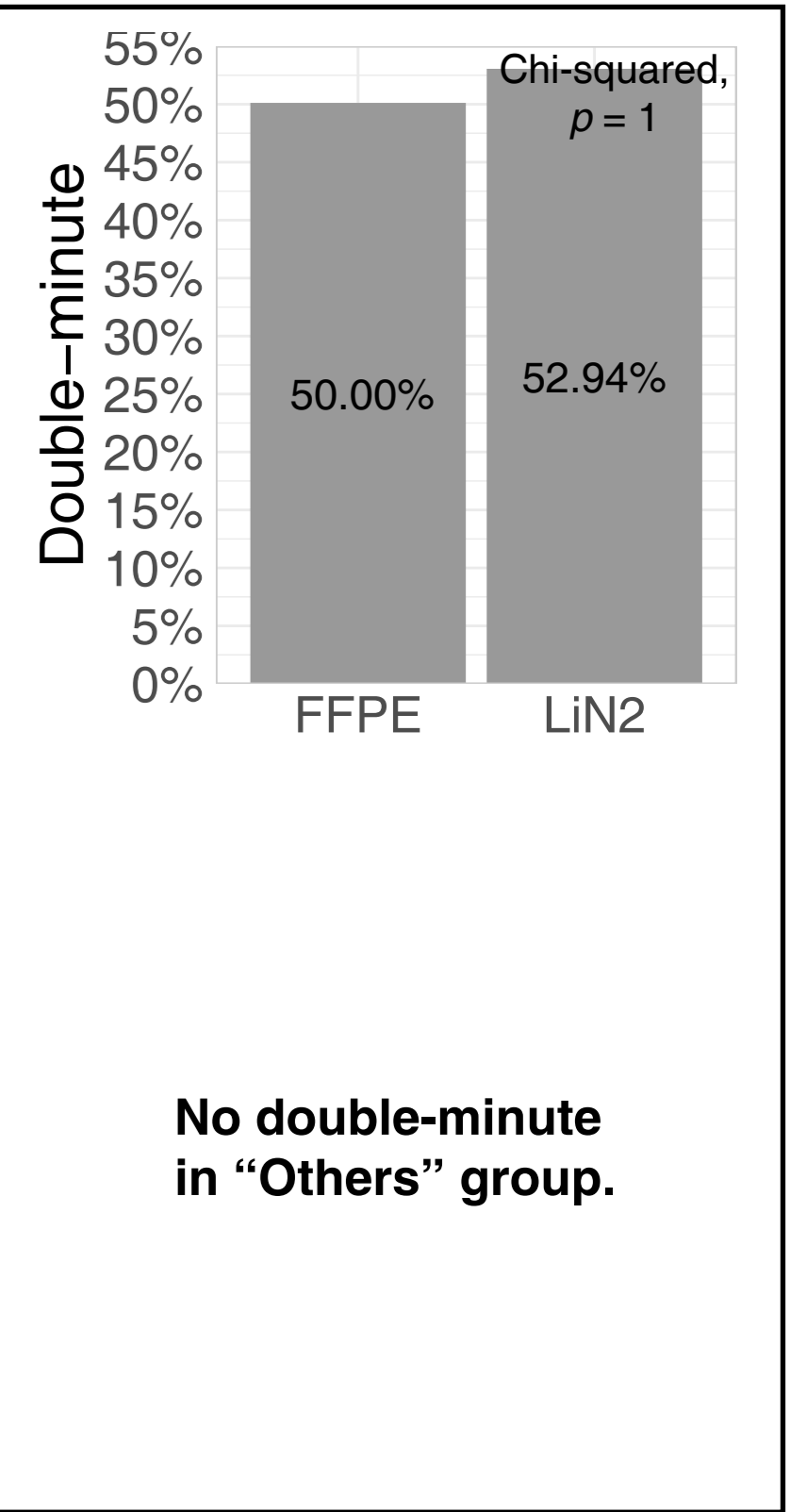

Others (n = 6)

A

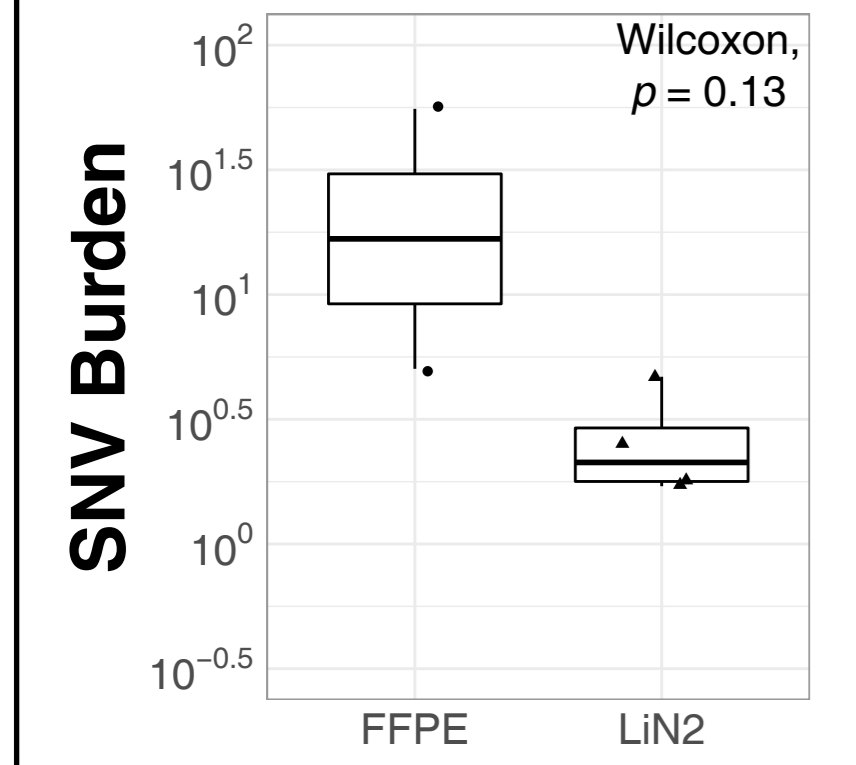

B

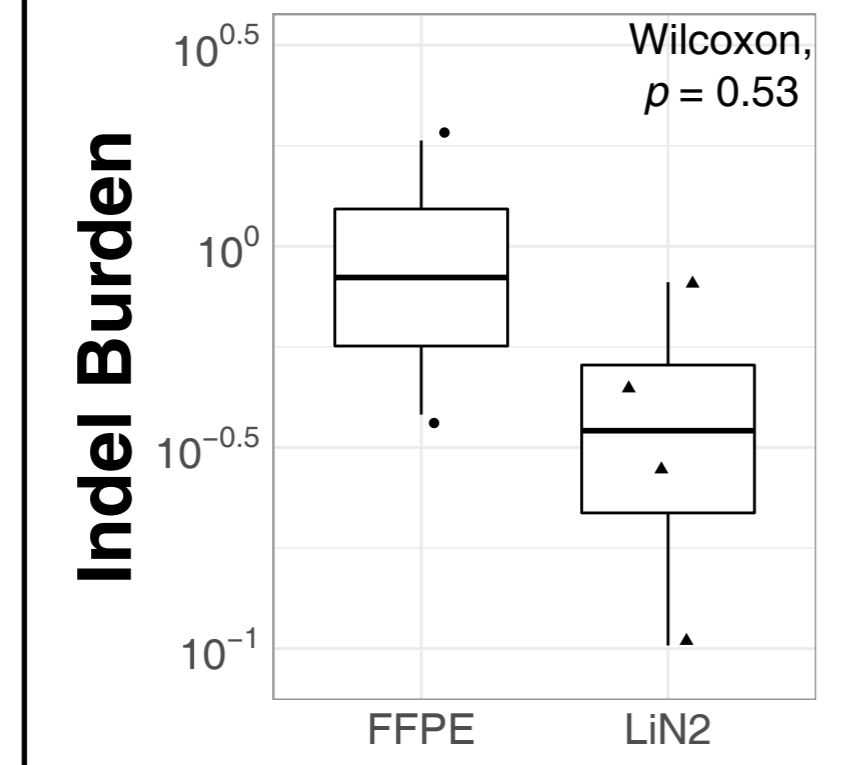

C

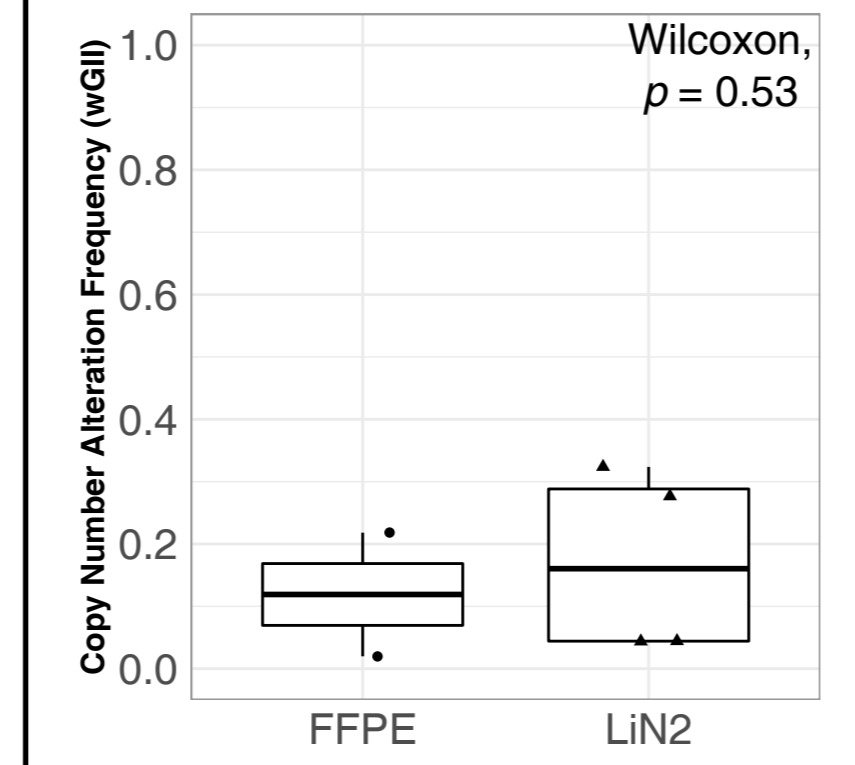

D

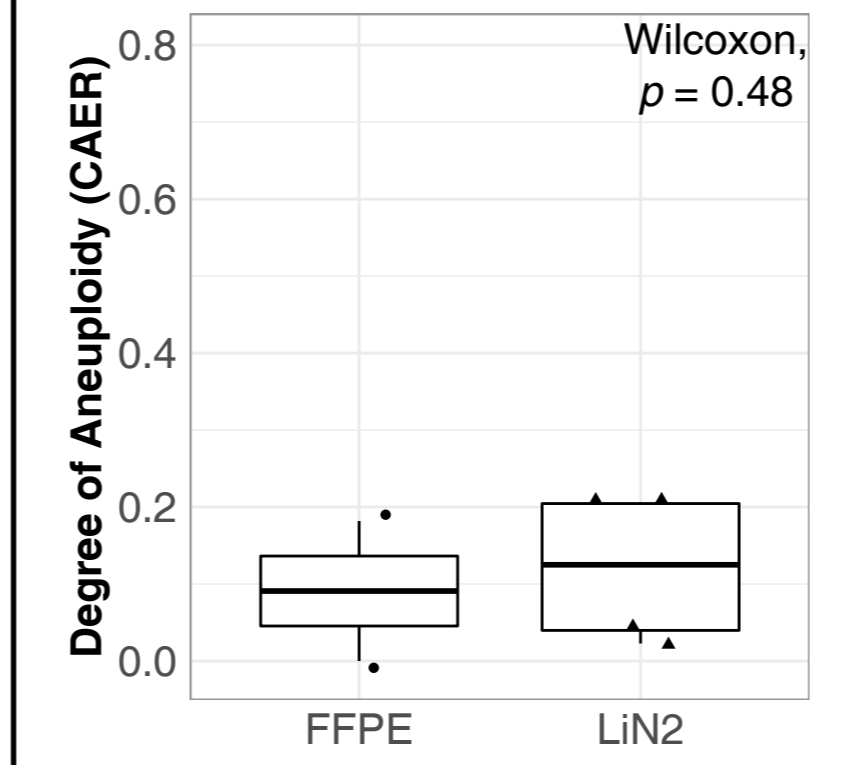

E

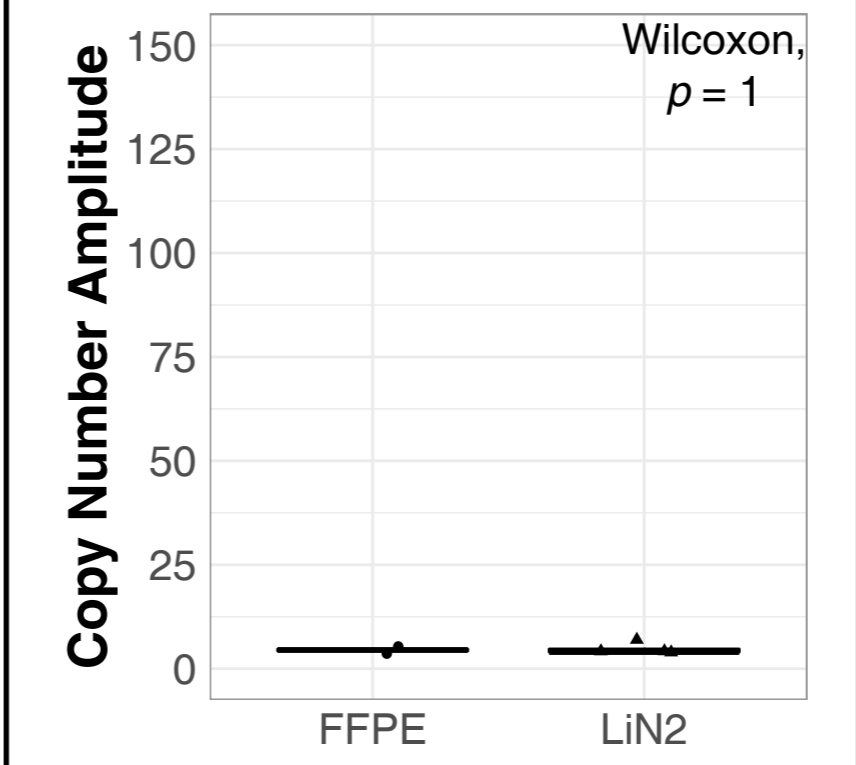

F

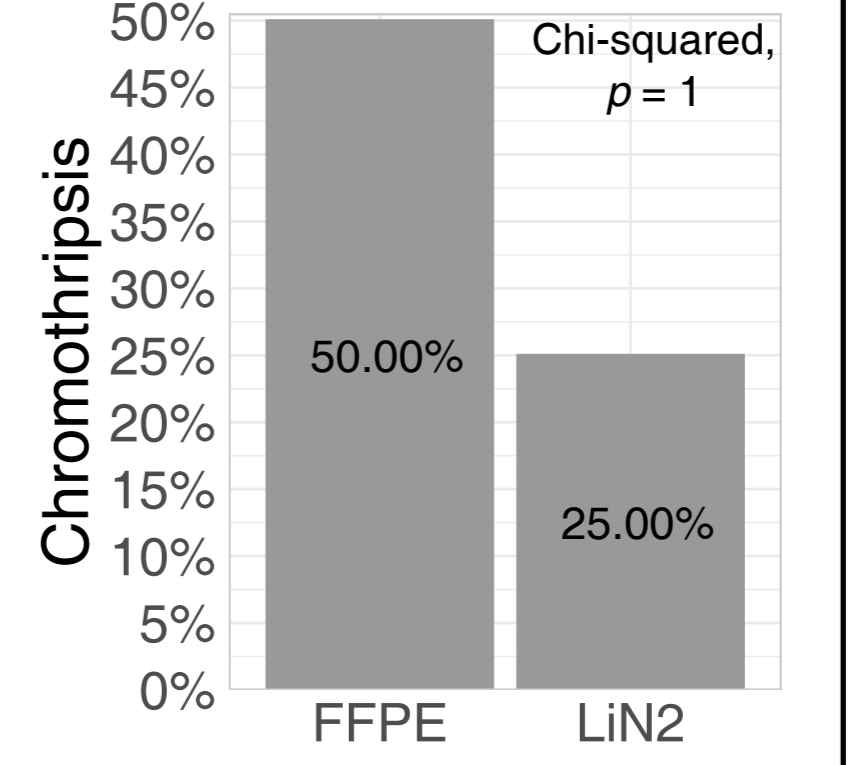

G

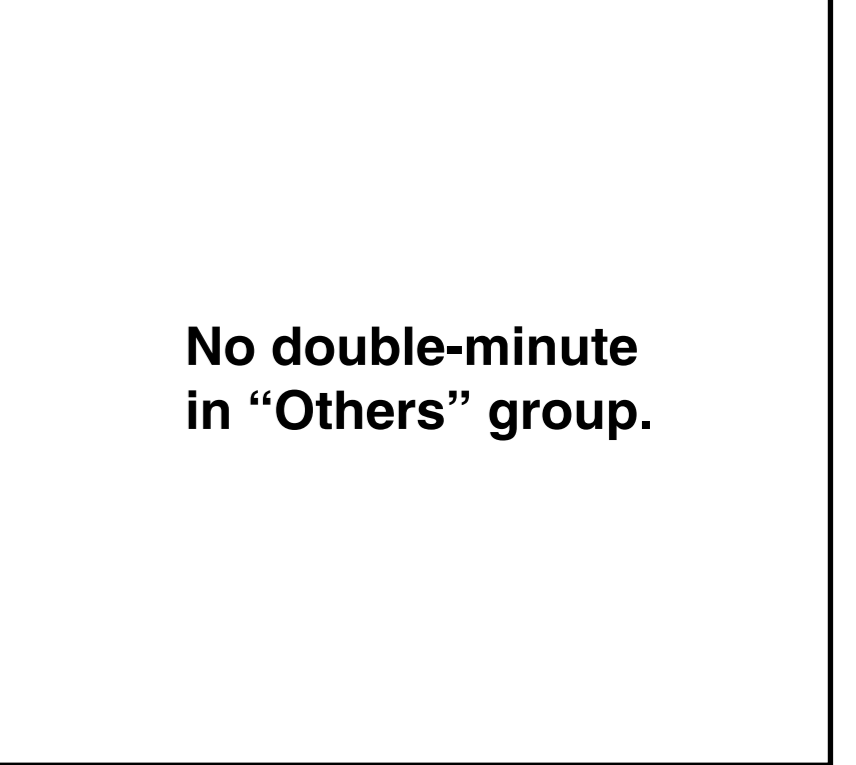

Supplement: Supplementary file 1 [file biomedicines-08-00574-s001.zip › biomedicines-966335 supplementary/Figure_S2.pdf]

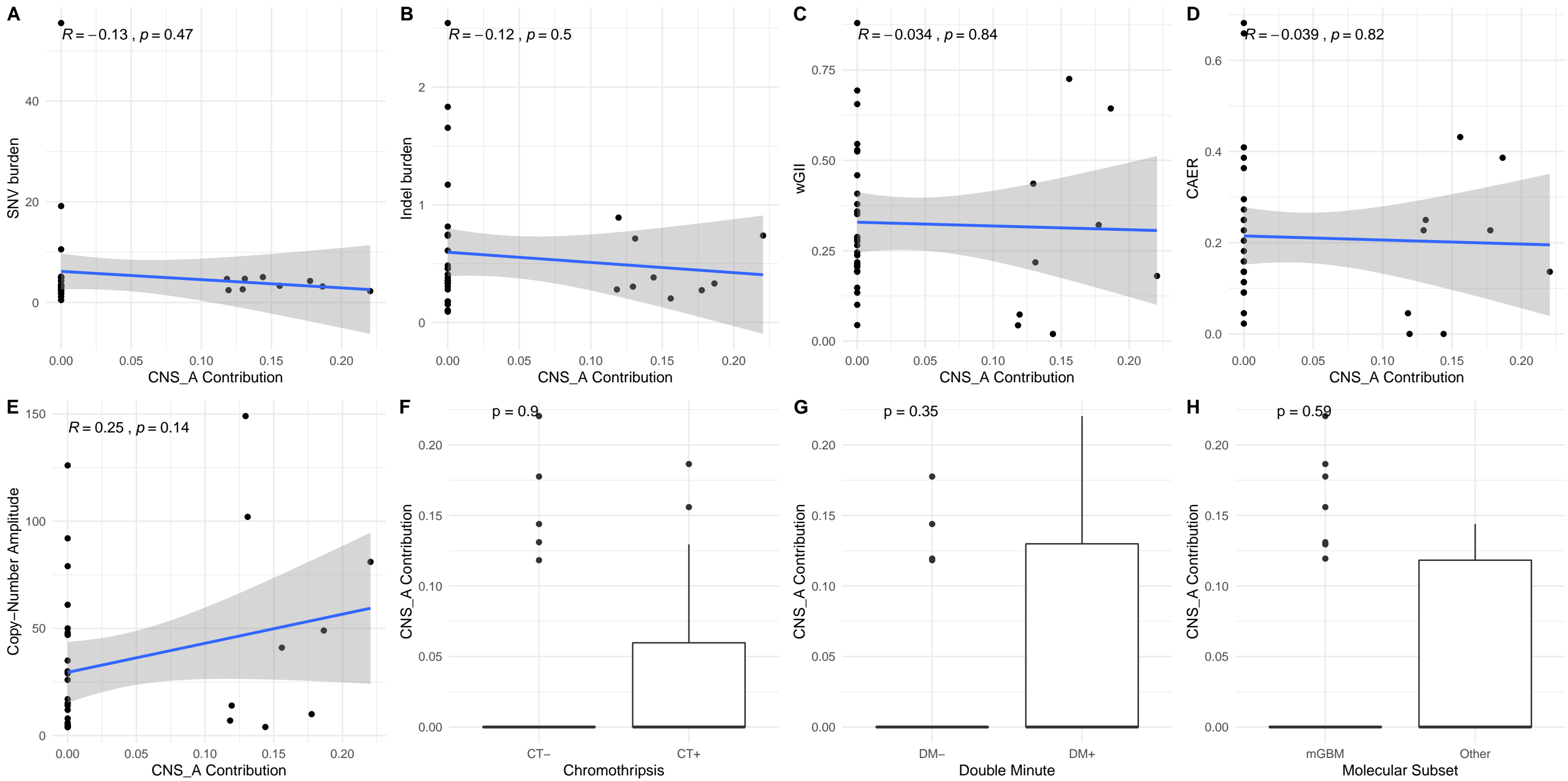

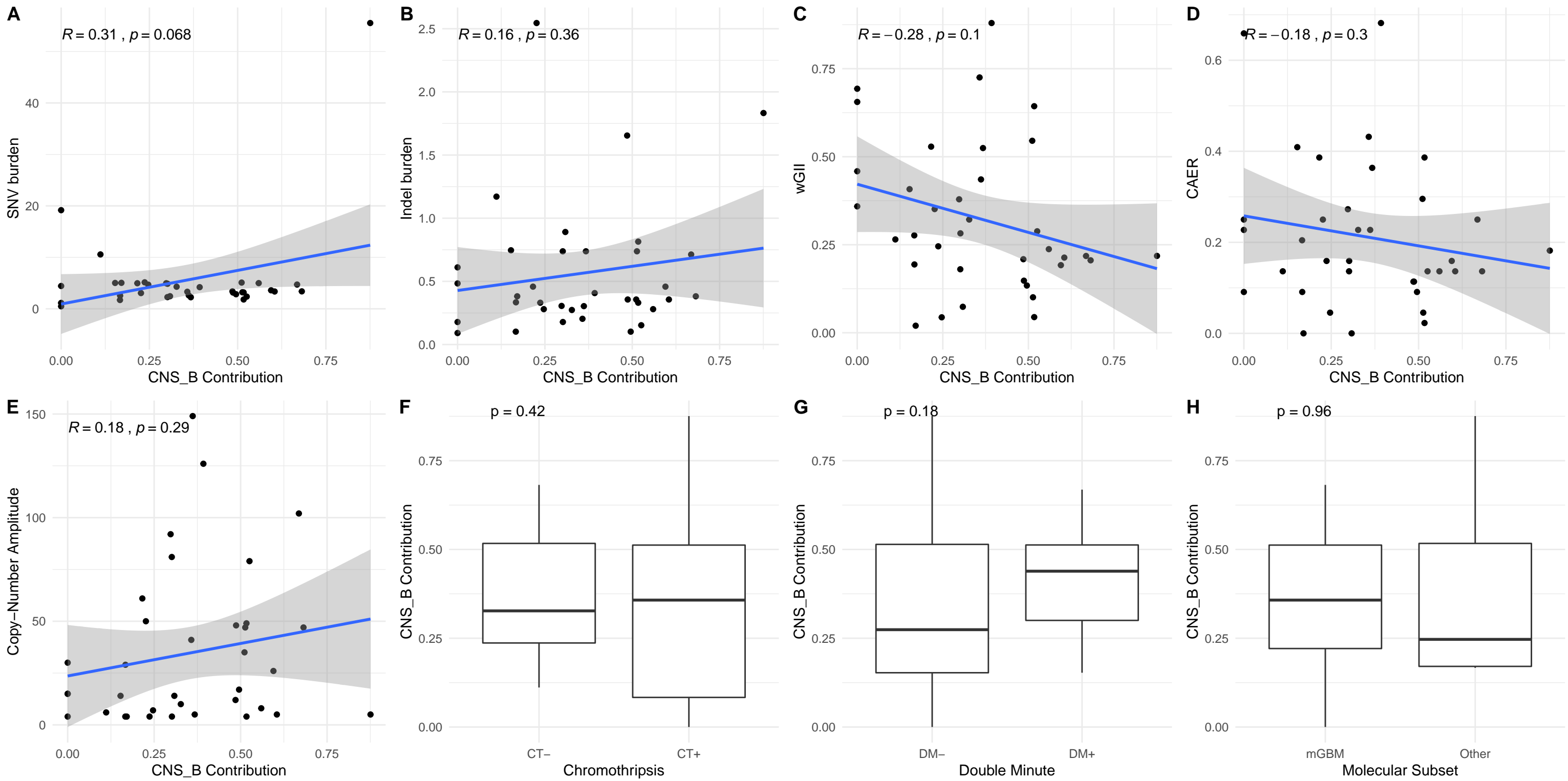

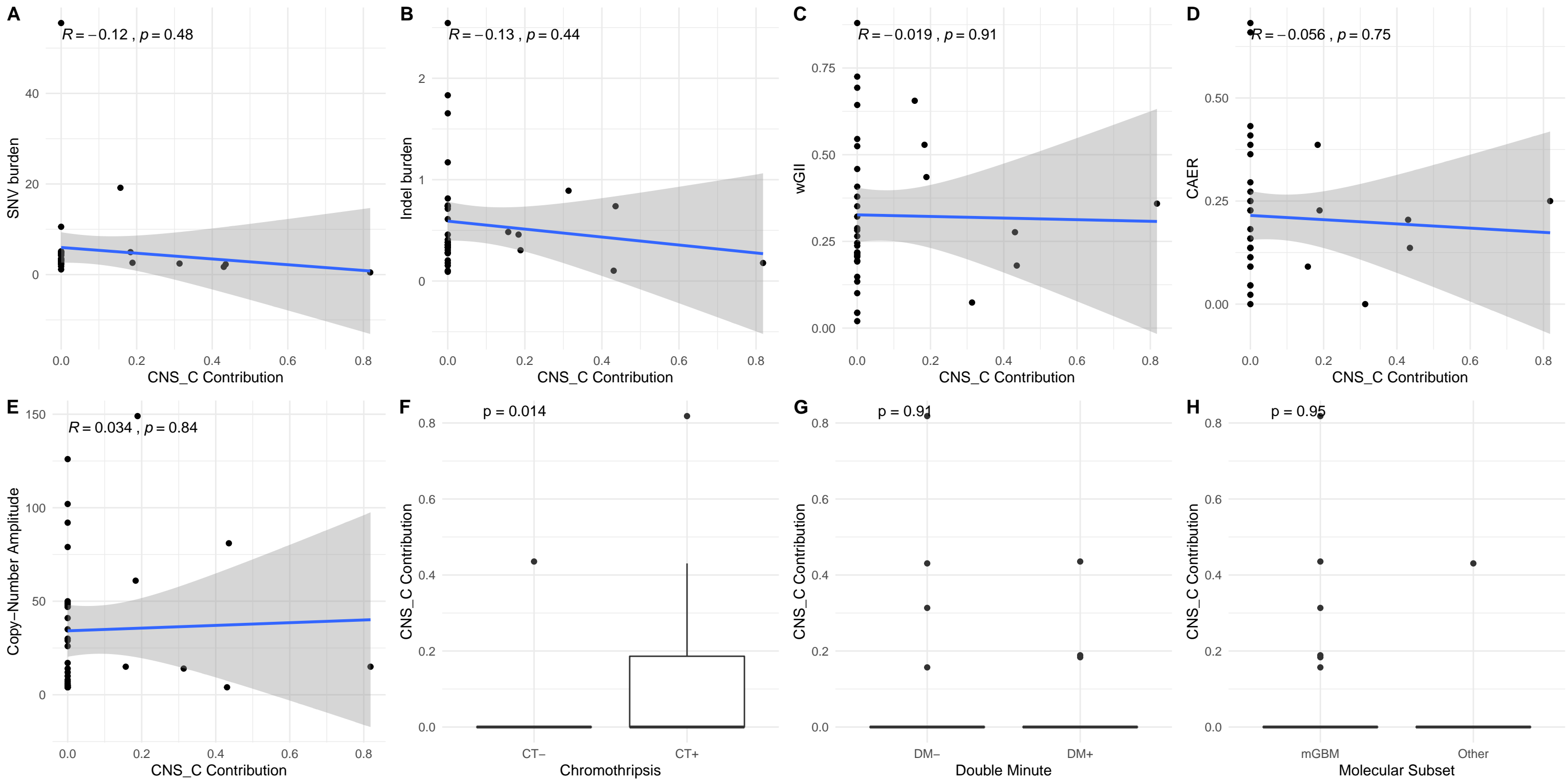

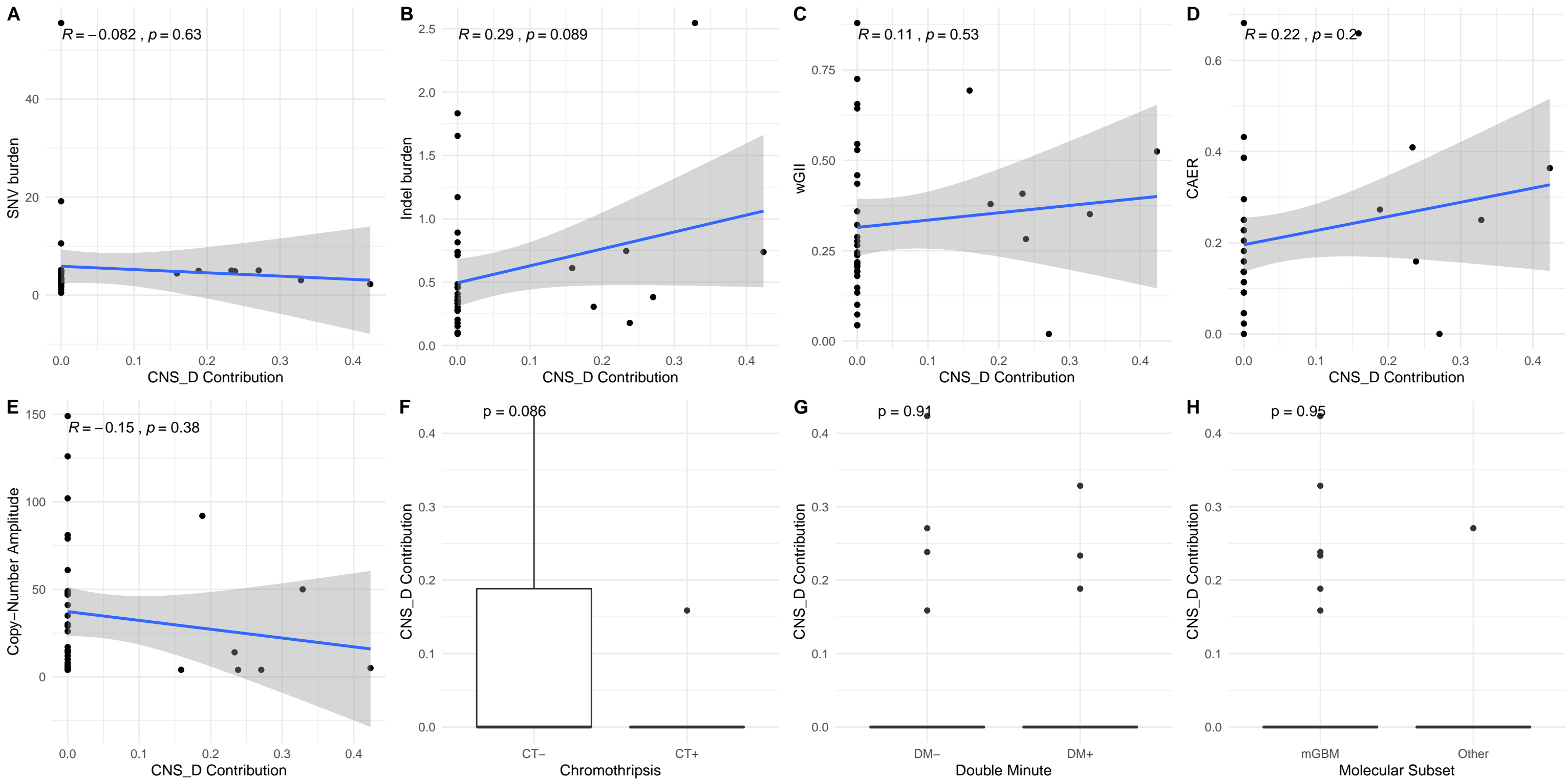

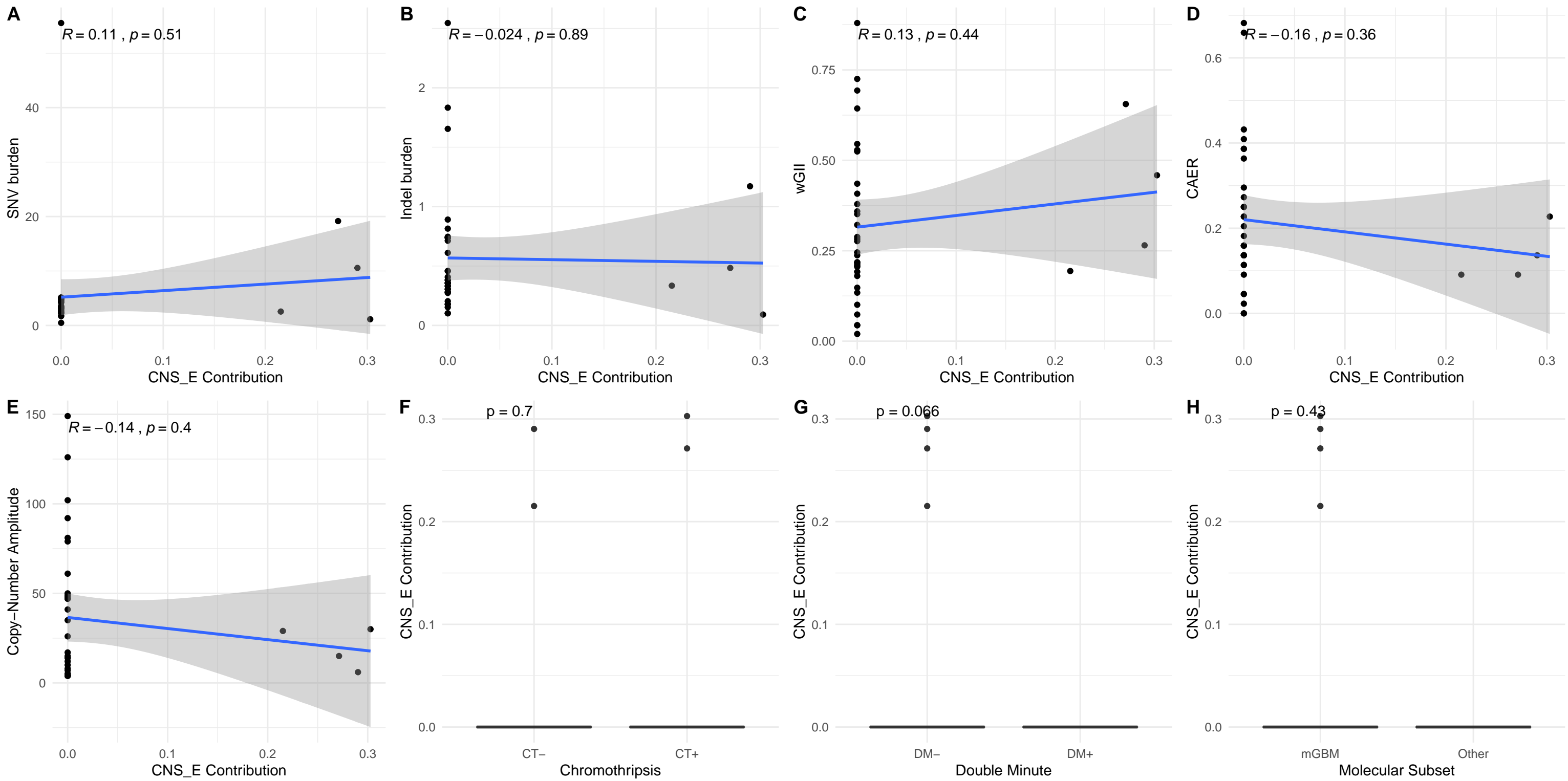

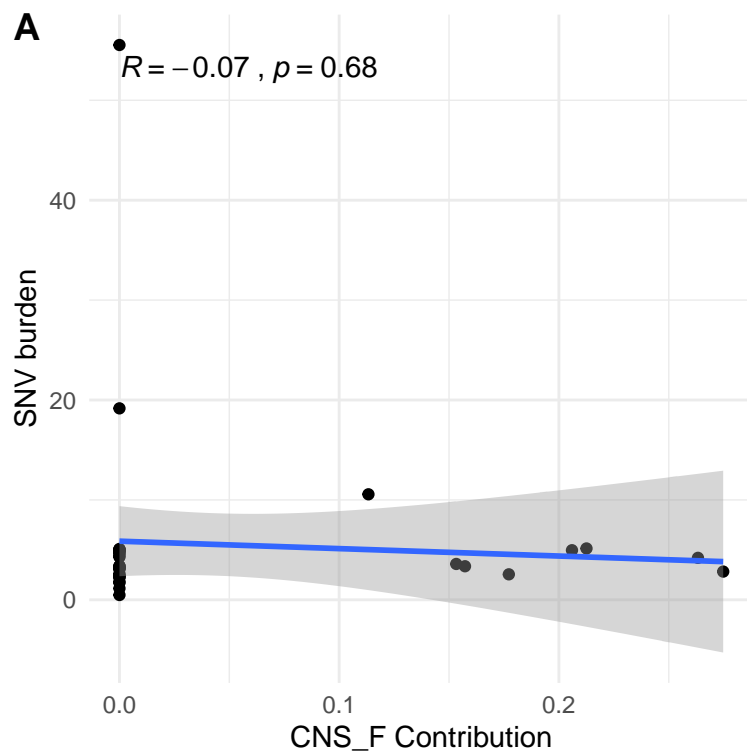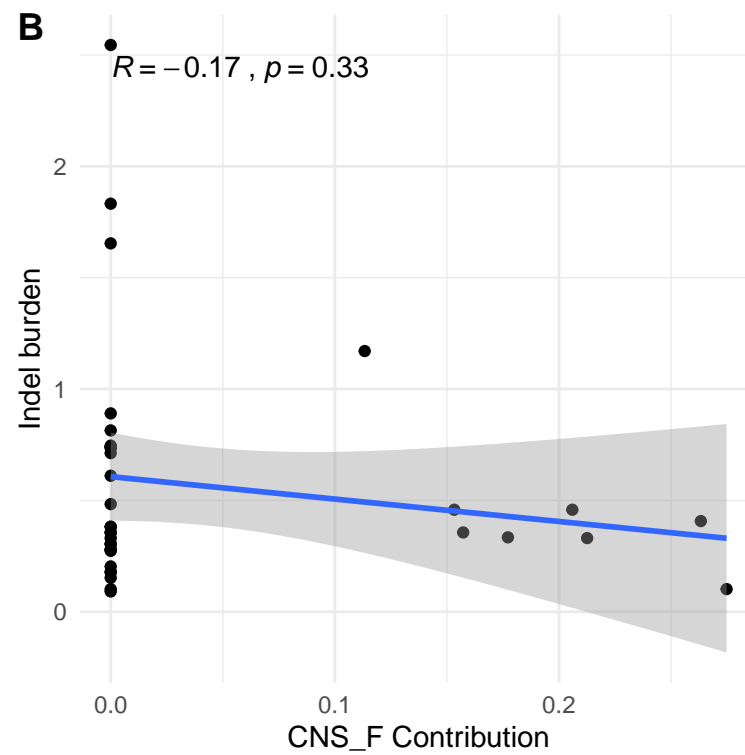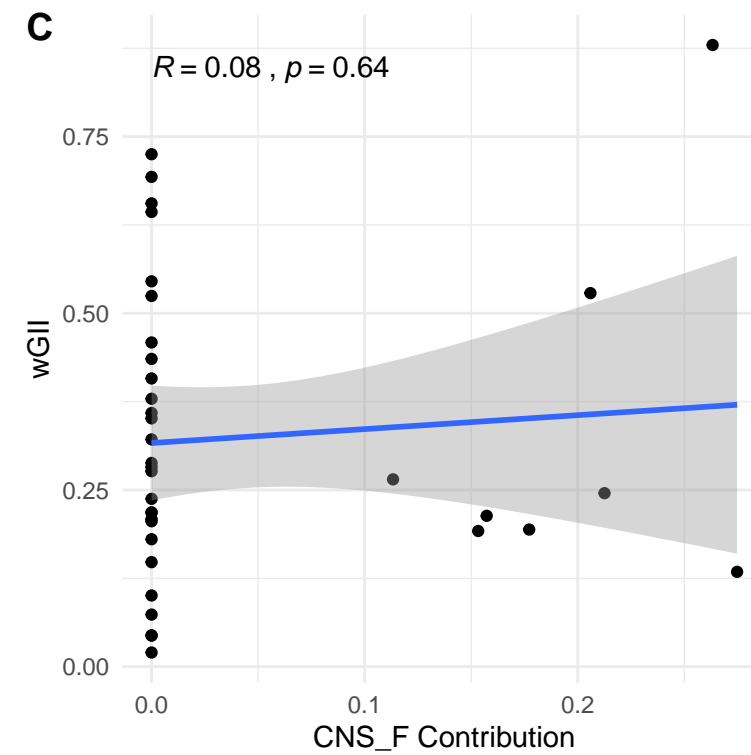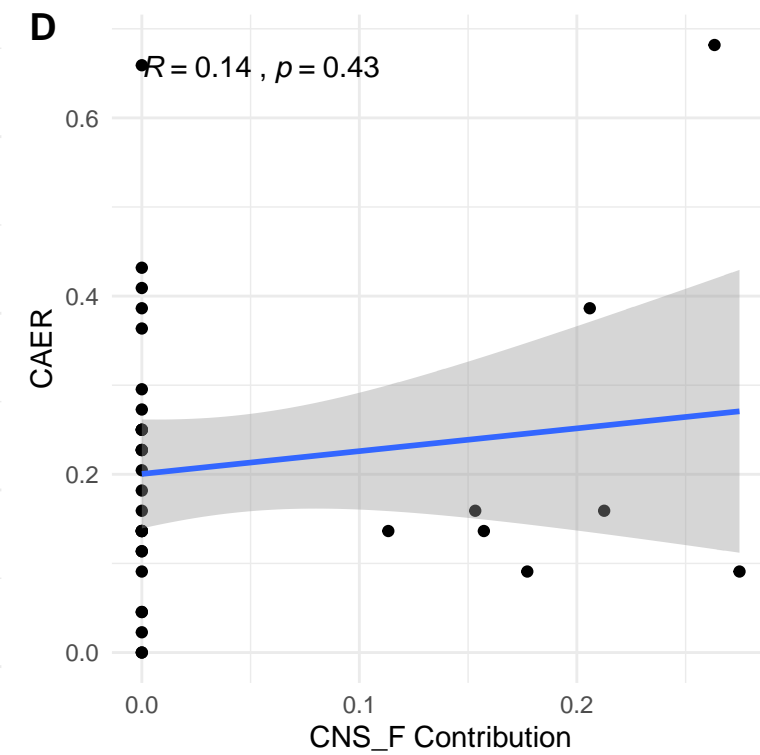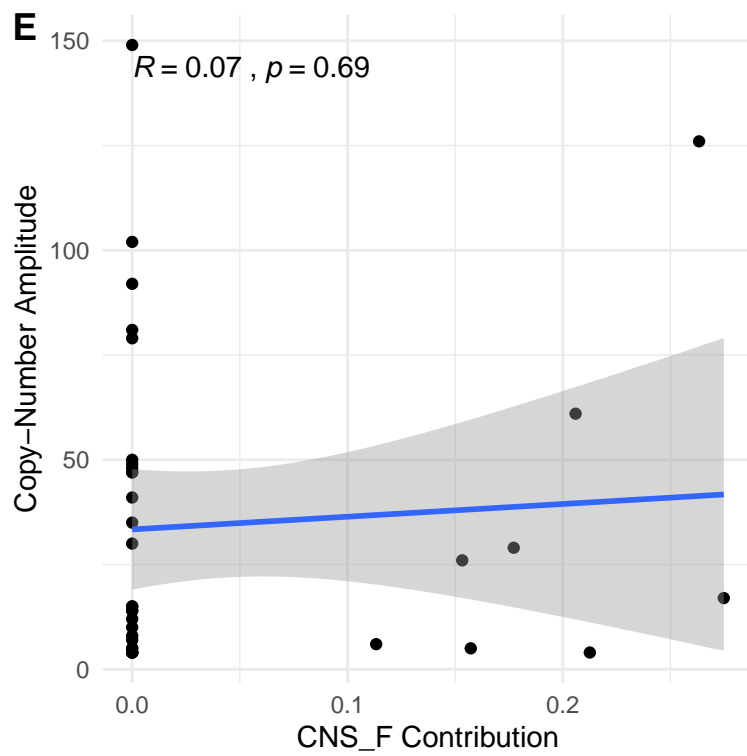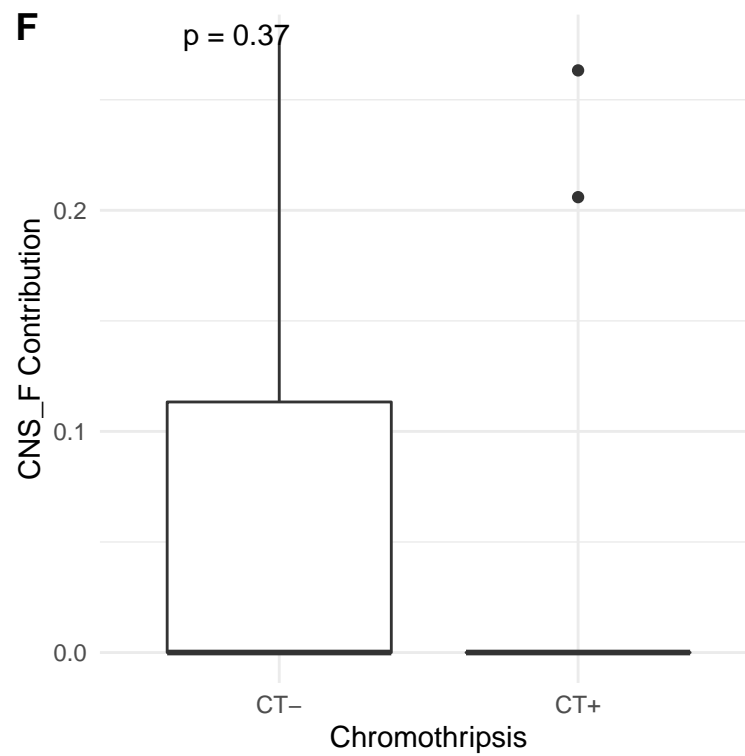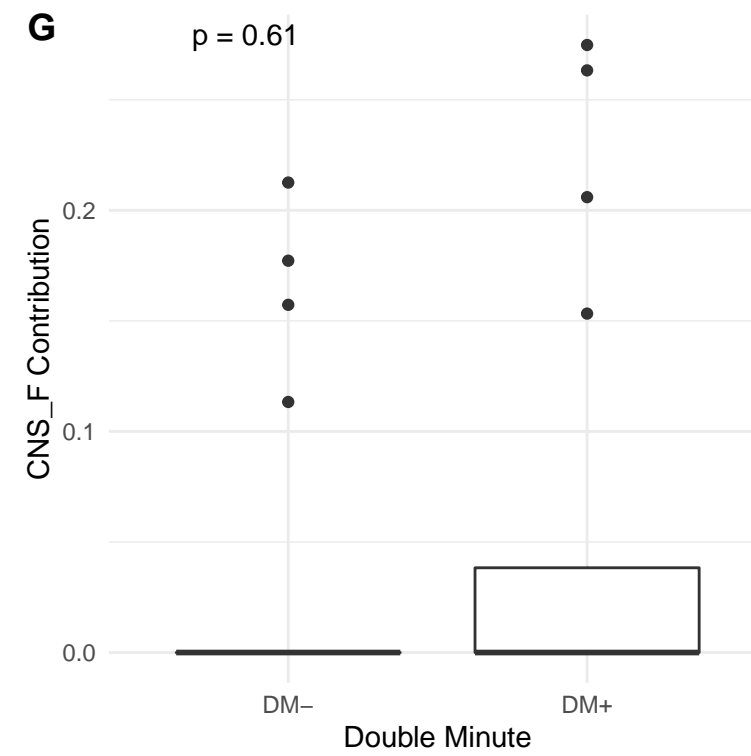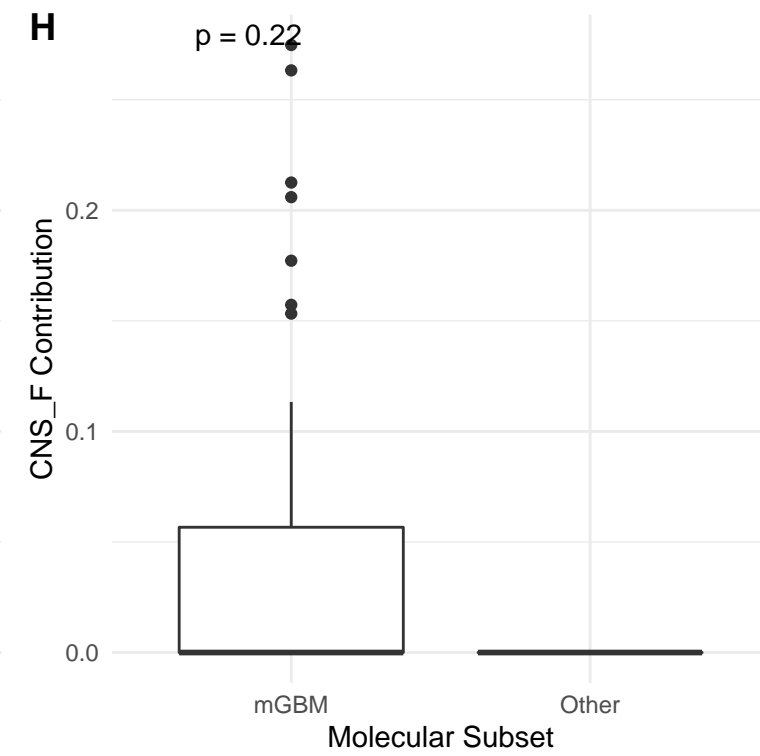

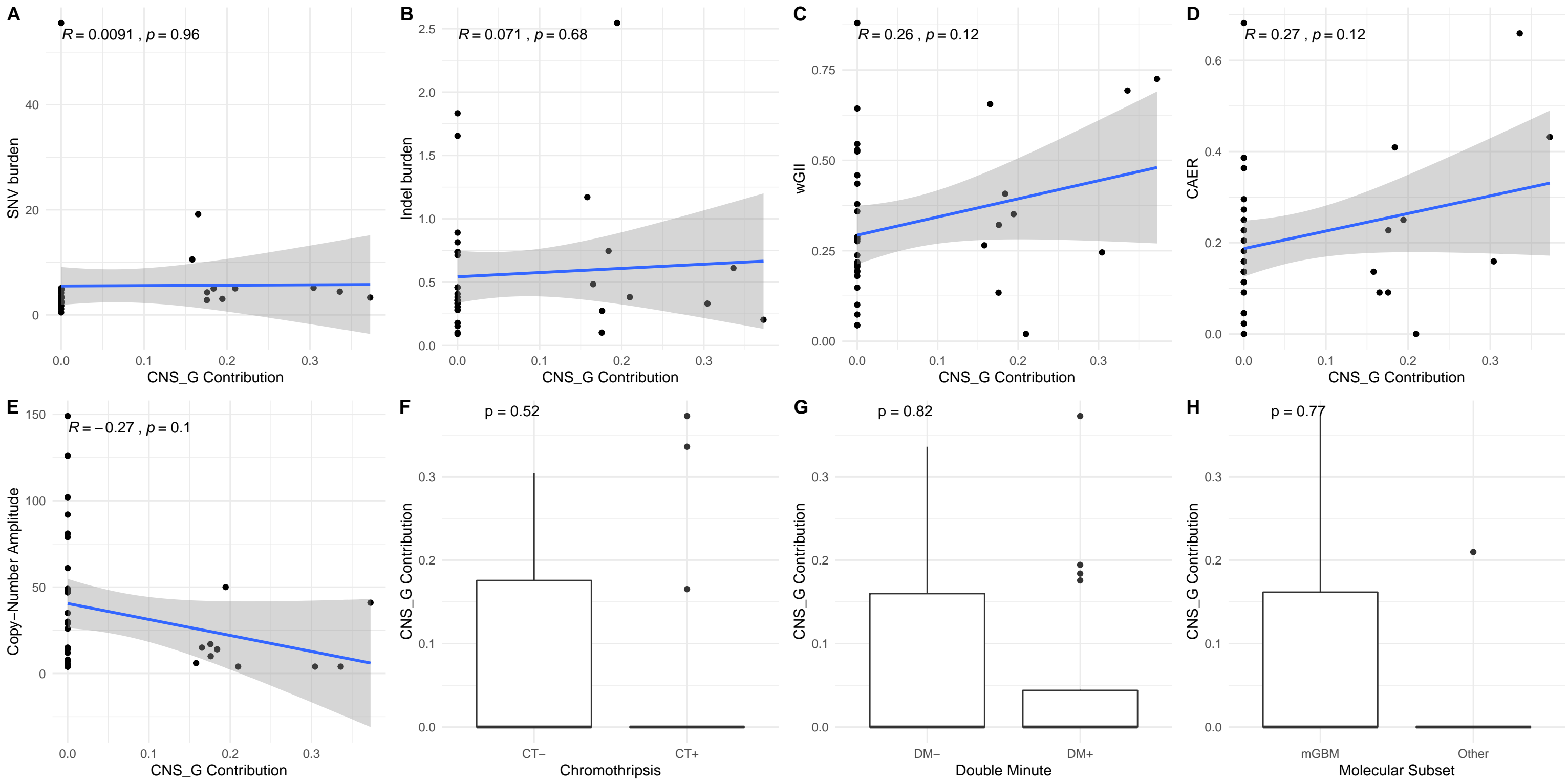

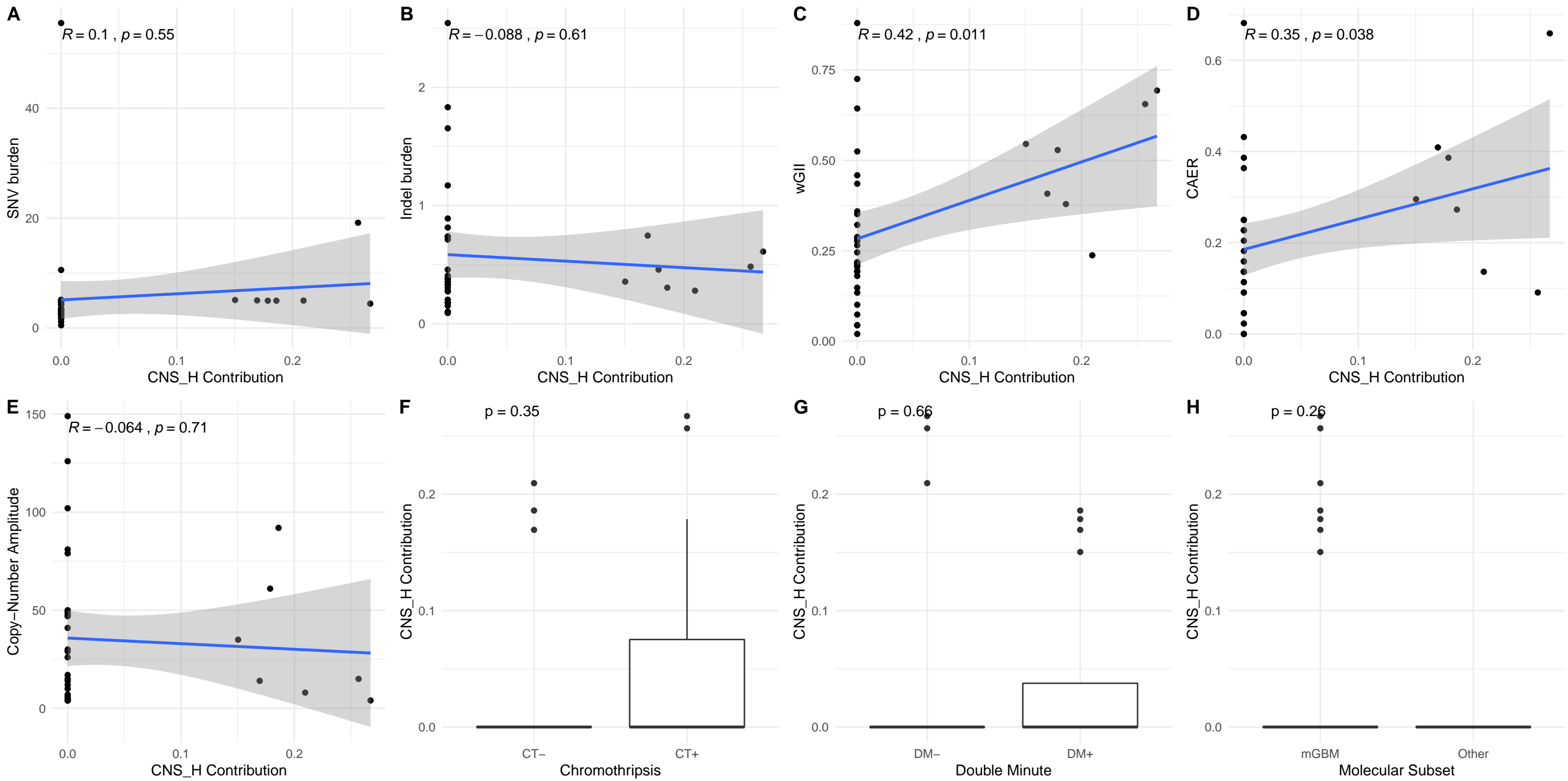

Supplement: Supplementary file 1 [file biomedicines-08-00574-s001.zip › biomedicines-966335 supplementary/Supplementary_Material_2.pdf]
